# Food-related worry and food bank use during the COVID-19 pandemic in Canada: results from a nationally representative multi-round study

**DOI:** 10.1186/s12889-023-16602-x

**Published:** 2023-09-05

**Authors:** Zachary Daly, Jennifer Black, Corey McAuliffe, Emily Jenkins

**Affiliations:** 1 School of Nursing, University of British Columbia, T201-2211 Westbrook Mall, Vancouver, BC V6T 2B5 Canada; 2 Faculty of Land and Food Systems, Food Nutrition and Health, University of British Columbia, 2205 East Mall, Vancouver, BC V6T 1Z4 Canada

**Keywords:** Food worry, Food banks, Food security, Covid-19 pandemic, Canada, Survey

## Abstract

**Background:**

Early in the COVID-19 pandemic, nearly one in five adults in Canada worried about having enough food to meet their household’s needs. Relatedly, throughout the pandemic, public messaging repeatedly urged Canadians to support food charities, including food banks. Yet few studies have examined food bank usage during the pandemic or whether food charities were widely used by Canadians worried about food access.

**Methods:**

This study draws on four rounds of nationally representative surveying conducted during the COVID-19 pandemic between May 2020 and December 2021 among adults 18 years and older living in Canada. Descriptive statistics were used to examine rates of food-related worry during all four survey rounds. Data from the fourth survey round, collected in December 2021, were used to explore use of food-based community programs since the onset of the pandemic, including food banks. Logistic regression analyses were used to examine differences in socio-demographic and health-related characteristics between adults who did and did not report accessing food banks before and after adjusting for household income.

**Results:**

Across survey rounds (*n* = 12,091), more than one in seven participants reported stress or worry related to having enough food to meet their household’s basic needs in the previous two weeks. Yet, by December 2021, fewer than 4% of participants reported ever accessing a food bank during the pandemic. Younger age, living with a child, financial concerns due to the pandemic, two different measures of food worry, pre-existing mental health conditions, disability, LGBT2Q + identity, and racialized or Indigenous identity, were each statistically significantly associated with higher odds of using food banks even when controlling for household income.

**Conclusions:**

Despite persistently high rates of food-related worry in 2020 and 2021 in Canada, relatively few adults reported accessing food banks or other charity-based community food programs. While respondents facing social, financial, and health-related inequities and reporting food worry were more likely to use food banks, most respondents did not report food bank use, regardless of financial or demographic circumstances or experiences of food worry. Findings align with previous research indicating that more adequate and comprehensive supports are needed to alleviate food-related-worry in Canada.

**Supplementary Information:**

The online version contains supplementary material available at 10.1186/s12889-023-16602-x.

## Background

Since the onset of the COVID-19 pandemic in early 2020, there has been renewed interest from researchers, health professionals, policy makers, and the public in understanding the experiences of individuals who live in food insecure households [1–6]. In Canada and the United States, household food insecurity is often defined as inadequate or insecure access to food due to financial constraints, and is monitored by Statistics Canada and the US Department of Agriculture on national surveys using the Household Food Security Survey Module (HFSSM) [7, 8]. Food insecurity is conceptualized on a spectrum, with milder forms characterized by worry, anxiety, and/or uncertainty about access to food; whereas moderate experiences include compromises to quality and/or quantity of food; and the most severe forms include missed meals, reduced food intake or going without food for longer periods [9, 10]. Food insecurity is a well-documented public health challenge with consequences for mental health, dietary quality, chronic disease management and mortality risk [11–18].

While the consequences of the most severe forms of food insecurity are clear, researchers have now demonstrated that milder experiences of food insecurity are also associated with poorer health. Survey respondents who answer affirmatively to even one question on the 18-item HFSSM survey module (a coding classification termed ‘marginal food insecurity’ in the Canadian classification scheme), are more likely to experience poorer self-reported mental and physical health than those with zero affirmatives on food insecurity survey scales [19–21]. Emerging research further suggests that stress and worry about running out of food may act as an independent measure and predictor of wellbeing in its own right [22–25]. For example, worrying about access to food could act as a precursor to reductions in the quality or variety of foods consumed and an early predictor of more severe forms of food insecurity [26, 27]. Furthermore, worrying about food access can contribute to psychological stress and may promote a deepened sense of relative depravation in situations where an individual’s food access is perceived to be worse off than that of others [23]. Two recently published Canadian studies drawing on data from 2020 in the early months of the COVID-19 pandemic, found strong associations between measures of food-related worry and several mental health outcomes including anxiety symptoms [25], depressive symptoms [25] and suicidal thoughts and feelings [24].

Since 2020, income shocks following workforce layoffs, lost work hours, workers’ illnesses, and quarantine orders, all had the potential to affect income-related food worries; as did ensuing economic and supply chain consequences and the rapidly increased rates of food inflation that began in late 2021 in Canada [1, 5, 28–30]. Further, fears of viral transmission while purchasing food, as well as substantial grocery delivery service delays in the early phases of the COVID-19 lockdowns [31], may have contributed to worries about food access that were independent of income or socioeconomic status. School closures and changes to food programs resulting from social distancing measures could have also created additional barriers to accessing food for the subset of individuals who drew on community-based food programs in 2020/2021 compared to pre-pandemic times [2, 32, 33].

Although national food insecurity monitoring in Canada using the HFSSM includes an item that relates to worrying about running out of food before having money to purchase more, few national studies during, before or since the onset of COVID-19 specifically report the prevalence or trends related to this specific experience of food insecurity. Still, data from the Canadian Community Health Surveys [34], Canadian Income Surveys [29] and from Pepetone et al. [35], suggest that the overall prevalence of food insecurity remained high on national surveys collected during the first years of the pandemic with over or close to 6 million Canadians reporting food insecurity in 2019, 2020 and 2021 [29]. To date, few studies have specifically probed the independent role that food worry serves in the pathways between food insecurity and poor health. But emerging evidence from Han et al. [25] and McAuliffe et al. [24] coupled with evidence that even milder or ‘marginal’ experiences of food insecurity predict poorer health outcomes compared to those reporting food security [19, 23], suggest the need to better document and describe experiences that relate to food worry.

As we build our understanding of how patterns of food-related worry and insecurity changed over the course of the pandemic, there is also a need for more nuanced understanding of the policy and programmatic responses that intersected with food-related experiences. Public discourse and media coverage during the pandemic frequently highlighted governmental and private investments in charity-based food programs (e.g., [36–39]). In Canada, food banks are commonly described as charitable food programs that provide a few days’ worth of supplemental food for individuals and families to take home and prepare, typically at no cost, and framed as the primary approach for addressing the food needs of those experiencing food insecurity [40]. Such food programs are actively promoted by fundraising campaings from the charities themselves and were publically lauded by government officials during the pandemic [41–43]. As the pandemic progressed, many Canadians, alongside the federal and provincial governments, directed record-breaking levels of funding toward food charities with the hopes of filling gaps left by inadequate social benefits programs [44–46]. For example, between April 2020 and December 2021 the Government of Canada committed an unprecedented $330 million towards the Emergency Food Security Fund to support emergency food providers and charitable food organizations, including food banks and meal programs [47]. Several provinces, municipalities, and local agencies also funded and mobilized supports for food banks, including a $3 million emergency grant from the province of British Columbia in March 2020 to “buy and distribute food, pay employees and cover other costs essential to the delivery of their food programs” [48]. Other high profile fundraising campaigns are exemplified by the Canadian Broadcasting Corporation’s annual holiday food bank fundraiser, which yielded record-breaking donations in winter 2020 [46]. Similar dynamics of growth in the charitable food sector outside of Canada have been reported elsewhere, including in the United States, United Kingdom, and several European Union countries [49–53].

Despite the unprecedented infusion of public dollars, private donations, and media coverage in Canada highlighting the efforts of charity-based food programs to meet household food needs during the pandemic, there is little public oversight of charity-based food programs and no nationally representative public-use data to characterize the patterns of population-level usage of community-based food programs including food bank use. Estimates from before the pandemic suggest that less than a quarter of food insecure households in Canada reported drawing on food banks when income was limited [54, 55].

Among the few studies that have generated empirical evidence about food *worry* in Canada during the pandemic, existing analyses have focused on the associations between such worries with mental health outcomes during the early stages of the pandemic [24, 25]. Yet there remains little empirical evidence available covering later time points, or regarding the potential associations between food worry and food bank usage in the context of the pandemic. While there has been research into how food banks and other charity-based food programs have responded to the pandemic in localized regions of Canada such as Ontario [56], Manitoba [2] and British Columbia [57], these studies have not been nation-wide. Furthermore, despite frequent media and governmental reports suggesting the widespread use of charity-based food sources in alleviating the food access concerns of Canadians during the pandemic, few national studies have described overall food bank *usage* in Canada in the context of the COVID-19 pandemic. One valuable exception, by Men and Tarasuk [28], described the demographic, employment, and behavioural characteristics of Canadians associated with food insecurity, but drew from data from April–May 2020 in the early months of the COVID-19 pandemic. Moreover, while Food Banks Canada conducts annual assessments of the Canadian food bank sector, their 2020 report focused on services *providers*, as opposed to service users [58]. These data suggested that food banks likely saw an increase in demand in the early weeks of the pandemic before emergency income benefits arrived from federal government programs. Yet other factors may have reduced or stabilized usage later in the pandemic. While more recent reports from Food Banks Canada [59] have reported on service users, the data included only limited exploration of demographic characteristics from users within the Food Banks Canada network of organizations and does not include insight about Canadians who do not participate in food programming.

This study aims to address gaps in knowledge in the context of the COVID-19 pandemic by 1) describing levels of food worry in Canada during the pandemic at four time points, using two distinct measures 2) presenting nationwide Canadian data on the prevalence of usage of food banks and other food-related services and 3) examining differences in socio-demographic and health-related characteristics, including food worry, between adults who did and did not report accessing food banks. Such analyses are valuable for providing empirical evidence to examine the current public debates regarding the extent to which charity-based food programs were utilized and met the needs of Canadians who had challenges related to accessing food during the COVID-19 pandemic.

## Methods

This paper draws on data from four rounds of a multi-round, nationally representative cross-sectional monitoring survey study, ‘*Assessing the Impacts of COVID-19 on Mental Health’*, aspects of which have been previously published [24, 60, 61]. This study was conducted through a partnership between researchers at the University of British Columbia and the Canadian Mental Health Association (CMHA). Initial survey items were informed by work commenced by the Mental Health Foundation of the United Kingdom in the early months of the pandemic [62]. These items were guided by research on the outcomes of past pandemics as well as through a participatory process involving people with lived experience of mental health conditions. These items were then adapted for the Canadian context in collaboration with the CMHA. Survey items in later rounds were further modified based on results from previous rounds and emerging evidence needs. Survey questions are included as Supplementary Material.

The four rounds of data were collected in May 2020, September 2020, January 2021, and November–December 2021. Surveys were distributed online in Canada’s two official languages, English and French, by the polling vendor Maru/Matchbox, which manages the Maru Voice Canada panel consisting of approximately 125,000 adults living in Canada 18 years and older. Panel participants were recruited through direct email, with targeted sampling through affiliate community partners to increase inclusion of populations that may be difficult to reach via the Internet (e.g., older adults, racialized populations). Panel members were randomly selected and invited from the wider panel to participate in this study, with invitations stratified by census informed categories (i.e., age, gender, household income, province/territory) and supplemented by statistical weights provided by Maru/Matchbox to yield a nationally representative sample according to these characteristics. The response-to-invitation ratios of the four rounds were 32%, 36%, 36% and 30%, respectively. While participants were not excluded from completing the survey in multiple rounds, most contributed to only one round.

### Variables of interest

#### Measures of food-related worry

This paper uses two measures of food worry. The first measure was used in all four rounds of surveying asked, “Have you been stressed or worried about any of the following as a result of the COVID-19 pandemic in the past 2 weeks?” with one option being “having enough food to meet my household’s basic needs”. Respondents could select “Yes”, “No”, or “Don’t know/Not applicable/Prefer not to say”. This question is broad and well-suited to capturing food worry stemming from multiple and interrelated concerns beyond finances. Examples of such concerns that are relevant given the context of the pandemic could include concerns about supply chain issues, challenges accessing services and risks or fears related to virus transmission in public spaces, including food stores [63]. Given the 2-week reference period, the item is well-suited to identify current or recent experiences relate to food worry. Notably, this item has been reported on in the United Kingdom [64], and its association with mental health outcomes has been reported on elsewhere [24]. After analyzing the first round of the survey collected in May 2020, the study team recognized the importance of more carefully examining food worry, and specifically, food worry related to insufficient income [10]. As such, in round 2, we added a second measure, drawn from the validated Household Food Security Survey Module (HFSSM), an annual survey led by the Government of Canada to monitor rates of food insecurity, specifically focused on income-related food worry [37]. This question asks whether: “You and other household members worried that food would run out before you got money to buy more. Was that often true, sometimes true, or never true in the last 12 months?” The response options included:** “**Often true; Sometimes true; Never true; Don’t know/prefer not to answer”. In addition to focusing more specifically on food worry that is related to finances, this item refers to a wider time frame (12 months) compared to the initial food worry question. Hence, for rounds 2–4 of the survey, there are two related, but distinct questions capturing experiences related to food worry.

#### Food bank and other food resource usage

To assess use of charitable food programs and other resources, all participants in all four rounds were asked “Since the onset of the COVID-19 pandemic and related restrictions in Canada, have you or any members of your household accessed food-based community programs to get food?” Response options were: “Food bank; Soup kitchens/Free meal programs; Meal or food programs from a school; Community kitchen program; Community Garden; Food voucher program (e.g., receiving gift cards for food from a charitable organization); Food delivered by a community program; Asking friends or family for help with food; or Other (unspecified community programs)”.

#### Socio-demographic and health-related characteristics

Socio-demographic variables included gender, age in years, region of residence (province/territory), highest education completed, annual household income, experiences of financial concerns, parent/guardian status, racialized/non-racialized status, and LGBT2Q+ identity (lesbian, gay, bisexual, transgender, two-spirit, queer, etc.). Several health and well-being-related questions were also included to identify participants living with a disability, a pre-existing (prior to the COVID-19 pandemic) mental health condition, or who had tested positive for COVID-19.

### Data analysis

Across analyses, *p*-values < 0.05 were considered statistically significant. All analyses were performed using SPSS version 28 using survey weights provided by Maru/Matchbox, to make the sample representative by region, sex, household income, and age according to Statistics Canada data from the 2016 Census of Population [65]. Descriptive statistics and percentages were generated to examine the prevalence of food-related worry and food bank and community-food program usage. A two-step approach, using logistic regression, examined associations between food bank usage by socio-demographic and health-related characteristics and food-related worry using data from the fourth survey round. First, odds ratios and confidence intervals were generated from unadjusted logistic regression models. Next, the models were adjusted for the potentially confounding effect of household income. The logistic regression analyses were restricted to the final round of survey data as the food bank usage question queried usage “since the onset of the pandemic” up until December 2021 and therefore assessed usage in early phases of the pandemic covered by earlier rounds of surveying.

## Results

### Sample description and prevalence of food worry

A total of 12,091 respondents participated in this study (*n* = 3,000 round 1; *n* = 3,027 round 2; *n* = 3,034 round 3; *n* = 3,030 round 4) (see Table 1). As described above, the sampling methodology was such that when paired with survey weights, demographic characteristics related to gender, province or territory, age, and income were nationally representative. Across the four rounds, the proportion of individuals reporting a pre-existing mental health condition (i.e., prior to the pandemic) ranged from 17.9% (round 3) to 19.0% (round 4). The proportion of individuals reporting financial concerns due to the pandemic in the two previous weeks ranged from 30.1% (round 3) to 38.6% (round 2). The proportion of respondents who reported being caregivers to individuals under 18 ranged from 20.1% (round 4) to 29.3% (round 2).

**Table 1:** Sample characteristics across all four survey rounds

	Round 1 May 2020 (*N* = 3000)		Round 2 Sept 2020 (*N* = 3027)		Round 3 Jan 2021 (*N* = 3034)		Round 4 Nov-Dec 2021 (*N* = 3030)	
	N	%	N	%	N	%	N	%
**Gender**^**a**^								
Cisgender man	1492	49.7	1464	48.4	1479	48.7	1479	48.8
Cisgender woman	1485	49.5	1478	48.8	1512	49.8	1500	49.5
Non-cisgender	23	0.8	84	2.8	43	1.4	51	1.7
**Age (years)**								
18–34	840	28.0	838	27.7	839	27.7	845	27.9
35–64	1490	49.7	1510	49.9	1501	49.5	1461	48.2
65 +	670	22.3	678	22.4	694	22.9	724	23.9
**Province/region**								
Alberta	330	11.0	335	11.1	337	11.1	339	11.2
British Columba/Territories	390	13.0	392	13.0	398	13.1	391	12.9
Quebec	720	24.0	724	23.8	719	23.7	714	23.6
Ontario	1140	38.0	1147	37.9	1156	38.1	1157	38.2
Manitoba/Saskatchewan	210	7.0	211	7.0	214	7.1	222	7.3
Atlantic Provinces	210	7.0	218	7.2	210	6.9	207	6.8
**Highest education completed**								
High school or less	421	14.0	475	15.7	426	14.1	473	15.6
Some college or university	498	16.6	563	18.6	451	14.9	523	17.3
College or university graduate	2082	69.4	1989	65.7	2156	71.1	2034	67.1
**Annual household income**								
< $25 k	253	8.4	196	6.5	239	7.9	217	7.2
25 k to < 50 k	497	16.6	549	18.1	497	16.4	506	16.7
50 K to < 100 k	990	33.0	983	32.5	971	32.0	955	31.5
100 k +	1260	42.0	1252	41.3	1236	40.7	1216	40.1
Don't know/prefer not to say	0	0.0	47	1.6	91	3.0	135	4.5
**Financial concerns in previous 2 weeks due to the pandemic**^**b**^								
Yes	1122	37.4	1168	38.6	912	30.1	920	30.4
No/don't know/not applicable/prefer not to say	1878	62.6	1859	61.4	2122	69.9	2110	69.6
**Parent/guardian with child under 18**								
Yes	645	21.5	886	29.3	647	21.3	609	20.1
No	2355	78.5	2141	70.7	2387	78.7	2421	79.9
**Race/ethnicity**^**c**^								
Racialized (non-Indigenous)	859	29.8	790	27.6	748	25.9	812	28.0
Indigenous	90	3.1	88	3.1	101	3.5	115	4.0
Non-racialized	1938	67.1	1982	69.3	2035	70.6	1971	68.0
**LGBT2Q + **								
Yes and unsure	269	9.0	270	8.9	270	8.9	276	9.1
No and prefer not to answer	2731	91.0	2757	91.1	2764	91.1	2754	90.9
**Identify as someone with pre-existing mental health condition**								
Yes	568	18.9	573	18.9	543	17.9	575	19.0
No and prefer not to answer	2432	81.1	2454	81.1	2491	82.1	2455	81.0
**Identify as someone with a disability**								
Yes	299	10.0	342	11.3	303	10.0	335	11.1
No and prefer not to answer	2701	90.0	2685	88.7	2731	90.0	2695	88.9
**Tested for COVID-19 and had a positive result**								
Yes	8	0.3	92	3.0	39	1.3	88	2.9
No/don’t know/prefer not to answer	2992	99.7	2935	97.0	2995	98.7	2942	97.1
**Stressed or worried about having enough food to meet my household’s basic needs as a result of the COVID-19 pandemic in the past 2 weeks?**								
Yes	525	17.5	602	19.9	431	14.2	477	15.7
No	2375	79.2	2284	75.4	2456	80.9	2408	79.5
Don't know/Not applicable/Prefer not to say	100	3.3	141	4.7	147	4.8	145	4.8
**Worried that food would run out before you got money to buy more in the last 12 months?**^**d**^								
Often true	-	-	198	6.6	102	3.4	122	4.0
Sometimes true	-	-	461	15.2	430	14.2	470	15.5
Never true	-	-	2308	76.3	2432	80.1	2368	78.1
Don’t know/prefer not to answer	-	-	59	2.0	70	2.3	71	2.3

^a^Round 1 respondents were asked “Which gender do you most identify with?”. Those who responded “Man” were classified as Cisgender men; those who responded “Woman” were classified as Cisgender women; and those who responded “Transgender woman/trans woman”, “Transgender man/trans man”, “Non-binary”, and “Two-Spirit”, were classified as Non-cisgender. This measure was altered in rounds 2–4, with participants instead asked which gender they most identify with alongside “What sex were you assigned at birth?”. Non-binary and transgender identities of participants were then determined by comparing responses to the two items

^b^E.g. going into debt, ability to pay bills, long-term economic impacts, etc.

^c^Participants were asked to report their ethnicity. Those who reported solely European origins were considered non-racialized, those who reported one or more non-European, non-Indigenous origins were considered racialized, and those who reported Indigenous origins were classified as Indigenous. Due to missing data this is not available for all participants

^d^This item was not asked in round 1

Note: Due to survey weights and rounding, numbers of cases may not always add up to N

The prevalence of reporting stress or worry about having enough food to meet respondents’ household’s basic needs in the previous two weeks due to the COVID-19 pandemic was higher than 1 in 7 in all survey rounds (17.5% in round 1, 19.9% in round 2, 14.2% in round 3, and 15.7% in round 4). The proportion of participants reporting that it was sometimes or often true that “you and other household members worried that food would run out before you got money to buy more” in the past 12 months, was 21.8% in round 2, 17.6% in round 3, and 19.5% in round 4 (this question was not asked in round 1).

### Food bank and community food program usage

When asked in round 4 whether respondents or any members of their household had accessed food-based community programs to get food since the onset of the pandemic, the majority of participants (91.4%, *n* = 2770) reported not accessing any of the food resources queried (see Table 2). While food banks were the most frequently reported community food resource used, by late 2021 less than 4% of respondents reported drawing on a food bank since the pandemic’s onset. Following food banks, the next most widely reported food resource utilized was asking friends or family for help with food (2.7%), followed by food delivered by a community program (1.9%), community gardens (1.2%), community kitchens (1.1%), and food voucher programs (1.1%). Fewer than 1% of participants reported accessing a soup kitchen/free meal program, or meal/food programs from a school or other unspecified food programs. Usage of these community-based food resources during the pandemic up to May 2020, September 2020, and January 2021, respectively is provided as a supplementary table. While we caution against making direct comparisons across survey rounds, owing to varied duration of timing covered by this question depending on the survey round, the main findings hold across survey wave. That is, regardless of survey round, the vast majority of Canadians had never accessed any of the food programs queried. And of those who did, fewer than 4% reported any food bank use, and fewer still accessed any of the other community-based programs queried.

**Table 2:** Use of community-based food resources since the start of the COVID-19 pandemic as reported in round four^ab^

	n	%
I haven't accessed any food programs	2770	91.4
Food Bank	113	3.7
Asking friends or family for help with food	83	2.7
Food delivered by a community program	59	1.9
Community Garden	37	1.2
Community Kitchen program	32	1.1
Food voucher program (e.g., receiving gift cards for food from a charitable organization)	33	1.1
Soup Kitchens/Free Meal programs	28	0.9
Meal or food programs from a school	21	0.7
Other	21	0.7

^a^November-December 2021, *N* = 3030

^b^Participants asked: "Since the onset of the COVID-19 pandemic and related restrictions in Canada, have you or any members of your household accessed food-based community programs to get food? (please select all that apply)"

### Associations between food bank use and participant characteristics

Reporting food bank use during the COVID-19 pandemic was statistically significantly associated with several socio-demographic and health-related variables, including age, household composition, educational attainment, household income and financial concerns, and testing positive for COVID-19 in unadjusted models (Table 3). Across the life-course, young (18-34y) [OR = 5.5; 95% CI 2.6–11.8] and middle age (34-64y) [OR = 3.8; 95%CI 1.8–8.1] adults were substantially more likely to report using a food bank compared to persons aged 65 years and older. Parents or guardians living with a child under 18 years of age were also more than twice as likely [OR = 2.3; 95% CI 1.5–3.4] to report food bank use compared to adults not living with children. Households reporting the lowest annual incomes (< $25,000 per year) were over 10 times more likely to utilize food banks compared to households reporting the highest income category ($100,000 + per year) [OR = 10.6; 95% CI 6.1–18.5]. Yet, adults with incomes between $50,000 to < $100,000 per year did not significantly differ in food bank usage compared to the highest income group. However, financial concerns in the previous two weeks due to the pandemic were also associated with higher odds of food bank use [OR = 4.7; 95%CI 3.2–7.0]. Compared to post-secondary graduates, respondents with high school or lower educational attainment were nearly twice as likely to report food bank usage in adjusted models [OR = 1.9; 95%CI 1.2–3.1].

**Table 3:** Food bank usage by participant characteristics since the start of the COVID-19 pandemic as reported in round four^a^

	Unadjusted models (odds ratio, 95% confidence interval)	Models adjusted for household income (odds ratio, 95% confidence interval)
**Gender**^**b**^		
*Cisgender man (n* = *1479)*	––	––
Cisgender woman (*n* = 1501)	0.97 (0.66–1.42)	0.87 (0.59–1.29)
Non-cisgender (*n* = 50)	1.77 (0.56–5.61)	1.13 (0.35–3.70)
**Age (years)**		
18–34 (*n* = 844)	**5.48 (2.55–11.77)**	**5.58 (2.57–12.08)**
35–64 (*n* = 1461)	**3.83 (1.80–8.14)**	**3.96 (1.85–8.49)**
*65* + *(n* = *725)*	––	––
**Province/region**		
*Alberta (n* = *340)*	––	––
British Columba/Territories (*n* = 391)	0.46 (0.21–1.00)	**0.45 (0.20–0.99)**
Quebec (*n* = 714)	0.7 (0.39–1.28)	0.60 (0.33–1.12)
Ontario (*n* = 1157)	0.64 (0.37–1.13)	0.66 (0.37–1.18)
Manitoba/Saskatchewan (*n* = 221)	0.93 (0.44–1.98)	0.86 (0.39–1.87)
Atlantic Provinces (*n* = 206)	**0.28 (0.08–0.91)**	**0.24 (0.07–0.80)**
**Highest education completed**		
High school or less (*n* = 473)	**1.91 (1.20–3.07)**	1.01 (0.61–1.67)
Some college or university (*n* = 523)	**1.80 (1.13–2.86)**	1.40 (0.87–2.27)
*College or university graduate (n* = *2034)*	––	––
**Annual household income**		
< $25 k (*n* = 217)	**10.6 (6.09–18.46)**	––
25 k to < 50 k (*n* = 506)	**3.81 (2.20–6.61)**	––
50 K to < 100 k (*n* = 956)	1.27 (0.70–2.31)	––
*100 k* + *(n* = *1216)*	––	––
Don't know/prefer not to say (*n* = 135)	0.41 (0.05–3.03)	﻿––
**Financial concerns in previous 2 weeks due to the pandemic**^**c**^		
Yes (*n* = 920)	**4.73 (3.18–7.02)**	**3.74 (2.49–5.63)**
*No/don't know/not applicable/prefer not to say (n* = *2110)*	––	––
**Parent/guardian with child under 18**		
Yes (*n* = 609)	**2.29 (1.54–3.40)**	**3.64 (2.37–5.60)**
*No (n* = *2421)*	––	––
**Race/ethnicity**^**d**^		
Racialized (non-Indigenous) (*n* = 812)	**1.75 (1.14–2.69)**	**1.63 (1.05–2.53)**
Indigenous (*n* = 115)	**6.58 (3.68–11.75)**	**5.20 (2.82–9.60)**
*Non-racialized (n* = *1971)*	––	––
**LGBT2Q + **		
Yes and unsure (*n* = 276)	**2.42 (1.48–3.94)**	**2.02 (1.22–3.34)**
*No and prefer not to answer (n* = *2754)*	––	––
**Identify as someone with a pre-existing mental health concern**		
Yes (*n* = 575)	**2.61 (1.76–3.97)**	**2.02 (1.34–3.03)**
*No and prefer not to answer (n* = *2455)*	––	––
**Identify as someone with a disability**		
Yes (*n* = 335)	**3.49 (2.29–5.34)**	**2.04 (1.29–3.22)**
*No and prefer not to answer (n* = *2695)*	––	––
**Tested for COVID-19 and had a positive result**		
Yes (*n* = 88)	**2.39 (1.09–5.23)**	2.22 (0.98–5.01)
*No, don’t know, and prefer not to answer (n* = *2942)*	––	––
**Stressed or worried about having enough food to meet my household's basic needs as a result of the COVID-19 pandemic in the past 2 weeks?**		
Yes (*n* = 477)	**6.85 (4.63–10.13)**	**4.75 (3.15–7.17)**
*No (n* = *2408)*	––	––
Don't know/Not applicable/Prefer not to say (*n* = 145)	1.5 (0.56–4.03)	1.07 (0.39–2.94)
**Worried that food would run out before you got money to buy more in the last 12 months?**		
Often true (*n* = 122)	**27.31 (15.47–48.21)**	**19.08 (10.51–34.64)**
Sometimes true (*n* = 471)	**11.9 (7.40–19.13)**	**8.94 (5.48–14.60)**
*Never true (n* = *2367)*	––	––
Don’t know/prefer not to answer (*n* = 71)	**3.78 (1.10–13.03)**	2.95 (0.84–10.36)

^a^November-December 2021, *N* = 3030

^b^Round 1 respondents were asked “Which gender do you most identify with?”. Those who responded “Man” were classified as Cisgender men; those who responded “Woman” were classified as Cisgender women; and those who responded “Transgender woman/trans woman”, “Transgender man/trans man”, “Non-binary”, and “Two-Spirit”, were classified as Non-cisgender. This measure was altered in rounds 2–4, with participants instead asked which gender they most identify with alongside “What sex were you assigned at birth?”. Non-binary and transgender identities of participants were then determined by comparing responses to the two items

^c^E.g. going into debt, ability to pay bills, long-term economic impacts, etc.

^d^Participants were asked to report their ethnicity. Those who reported solely European origins were considered non-racialized, those who reported one or more non-European, non-Indigenous origins were considered racialized, and those who reported Indigenous origins were classified as Indigenous. Due to missing data this is not available for all participants

^e^Reference category in italics, odds ratios which are significant at *p* < 0.05 are bolded

Note: Due to survey weights and rounding, numbers of cases may not always add up to N

Higher prevalence of food bank usage was reported among participants from members of equity deserving groups, including adults who identified as LGBT2Q+ [OR = 2.4, 95% CI 1.5–3.9]. Food bank usage was likewise reported by racialized (non-Indigenous) participants [OR = 1.8; 95%CI 1.1–2.7] and Indigenous participants [OR = 6.6, 95%CI 3.7–11.8] at higher rates than non-racialized participants. Furthermore, participants who reported a pre-existing mental health concern [OR = 2.6; 95%CI 1.8–3.9], having a disability [OR = 3.5; 95%CI 2.3–5.3], or testing positive for COVID-19 at any time during the pandemic [OR = 2.4; 95%CI 1.1–5.2] had higher rates of food bank use compared to those not reporting these identities and experiences. The relationships between the socio-demographic and health-related variables remained statistically significant, even after controlling for household income, apart from highest attained education and having tested positive for COVID-19 at any time, which were no longer significant after controlling for household income.

### Associations between food bank use and food worry

Respondents who reported being “stressed or worried about having enough food to meet my household’s basic needs as a result of the COVID-19 pandemic in the past two weeks” were more likely to report using a food bank compared to those who did not (12.6% vs 2.0%) [OR = 6.9 95% CI 4.6–10.1]. Similarly, those who reported sometimes [OR = 11.9; 95%CI 7.4–19.1] or often [OR = 27.3; 95%CI 15.5–48.2] worrying that food would run out before they got money to buy more in the last 12 months had significantly higher odds of reporting food bank use compared to those who did not. After controlling for the potentially confounding effects of household income, the magnitude of associations between food worry and food bank usage were diminished, but remained strong and statistically significant for those who reported being “stressed or worried about having enough food to meet my household’s basic needs as a result of the COVID-19 pandemic in the past two weeks” (yes versus no) [OR = 4.8; 95%CI 3.2–7.2] as well as for those who endorsed “sometimes true” or “often” true” (each versus “never true”) [OR = 8.94; 95%CI 5.48–14.60 and OR = 19.08; 95%CI 10.51–34.64 respectively] for “worrying that food would run out before they got money to buy more in the last 12 months”. While respondents who reported food-related worry were more likely to report food bank use than those who did not across all models, less than one in four respondents who reported often worrying that food would run out used a food bank at any point since the onset of the pandemic.

## Discussion

This study builds upon data collected in the early months of the COVID-19 pandemic [24, 25, 28, 29], confirming that persistently high proportions of adults living in Canada experienced stress or worry about having enough food to meet their household’s basic needs in 2020–2021. Using two measures of food worry and drawing on data collected at four time points between May 2020 and December 2021, we found that a considerable proportion of adults living in Canada experienced worry about having enough food within the past two weeks during the COVID-19 pandemic. By December 2021, approximately one in five adults reported that they or other household members sometimes or often worried that food would run out before they had money to buy more in the past 12 months. As we have reported elsewhere [24], drawing on data from the early months of the pandemic in Canada, food worry was more common among adults who were younger, Indigenous, parents/guardians living with children, reported low household income, had financial worry, or were living with a pre-existing mental health condition. This aligns with other research highlighting that unstable or insufficient access to food due to financial constraints is strongly associated with experiences of poverty and material deprivation, racial inequities, higher healthcare costs, poorer physical health outcomes and worse mental health [7, 24].

We cannot directly compare the current findings with rates of food-worry before the onset of the COVID-19 pandemic as one of the core food worry measures used here was novel and the other, which draws on a well-used survey item from the Household Food Security Survey Module, is seldom reported as a stand-alone estimate. However, these findings point to the potential value of monitoring and reporting food-related worry in its own right in future studies to understand the nuanced experiences that shape food insecurity and its consequences. We also note that national surveys using the HFSSM also typically ask about experiences over a 12-month reference period, and our first food worry estimate only referenced food worry in the past two weeks. Therefore, estimates generated here likely underestimated prevalence of episodic worries experienced across a longer timeframe in the early months of the pandemic.

Our findings align with and further extend a robust body of scholarly evidence that has repeatedly found that Canada’s social safety net was insufficient for mitigating the food-related worries and financial consequences of the pandemic for many adults living in Canada [8, 39]. In line with this, the coalition of researchers and practitioners known as the Global Solidarity Alliance (GSA) for Food, Health and Social Justice assert that food is a fundamental human right enshrined in the United Nations’ Declaration of Human Rights [40]. Drawing on international evidence from the past several decades, the GSA’s vision of improving outcomes for those experiencing and at-risk of food insecurity includes improved labor “policies ensuring workers are paid livable wages with universal income security and responsive government social protections programs available to all”. Such proposals are in line with the recommendations generated from national evidence-based recommendations on food insecurity from the Canadian PROOF group’s food insecurity research program which call for improved “policies that ensure all low-income households have enough money for food and other basic needs, regardless of their income source” [8].

Our results align with evidence from Men & Tarasuk’s analysis of the Canadian Perspectives Survey Series 2 data, which reported that by May 2020, less than 10% of respondents had made use of charity-based food programs during the early stages of the pandemic [28]. Food Banks Canada has reported that by March 2022, 13.9% of adults in Canada over age 18 had “accessed food or meals from a community organization since March 2020” [40]. This number differs from our own findings, in which 8.6% endorsed accessing any community-based food resources since the start of the pandemic, up to December 2021. However, we note that the Food Banks Canada survey was administered at a later date compared to our survey, and that Food Banks Canada utilized a different sampling strategy, based on automated phone interviews.

Canadian public discourse and legislative debates frequently focus on food charity and food banks specifically as a primary strategy to address food-access related challenges and insecurity [66–68]. Upon announcing historically large investments to the COVID-19 Emergency Food Security Fund in October 2020, the Prime Minister of Canada’s office asserted that “by supporting Canada’s food banks and local food organizations, we are making sure vulnerable Canadians can get the food they need, when they need it most” [41]. Yet, our findings run contrary to claims that food banks were readily utilized by those facing financial instability or barriers to meeting their food-related needs during the pandemic. Respondents were more likely to report food bank usage if they experienced food worry, even after adjusting for household income. Yet, we still found that only a small minority of the individuals who reported food worry, individuals who ostensibly may have benefited from improved food-related supports, ever used a food bank during the pandemic. Notably, this finding was robust to two distinct measures of food worry.

Our results build on existing Canadian evidence documenting many limitations of charitable food programming generated before the COVID-19 pandemic [54, 67, 69, 70]. The finding that relatively few participants in our surveys reported drawing on food-based community programs of any kind, and that the majority of individuals reporting food worry did not access food banks since the onset of the pandemic, is perhaps not surprising given the many well-documented obstacles to using food-based community programs before and during the pandemic [67, 71–73]. For example, commonly reported barriers to food bank use include: lack of access to services due to place of residence, lack of transportation or time, long wait times, stigma due to public line-ups, stressful environments, and suboptimal quantity and quality of food to meet dietary needs or preferences [67, 69, 72, 74–76]. Slater et al. further documented several specific and substantial impacts of COVID-19 on the charitable food sector in the Canadian province of Manitoba that likely exacerbated the challenges for those running and relying on charitable food programs [2]. Additional pandemic-specific challenges included: increased demand for services; food supply challenges; and difficultly in adequately staffing and supporting the human resource needs of programs. This included the challenges of managing the emotional stressors and vulnerabilities among staff, volunteers and clients while also overseeing complex and evolving changes to service delivery, safety protocols amid insufficient resources and supports to meet the needs of clients [2]. Other challenges to accessing and running food banks in the context of the pandemic have also been reported in Ontario [56], British Columbia [57] and by Food Banks Canada [59].

This study illustrates that overall food bank usage remained relatively low during the pandemic in Canada, but there were notable socio-demographic and health-related characteristics that distinguished service users. For example, the odds of accessing food banks were significantly higher for younger adults, particularly those aged 18–34, and groups who were likely disproportionately impacted by labor market conditions and the economic fallout from the pandemic, particularly those with the lowest incomes [77]. Racialized and Indigenous adults, those with lower educational attainment, and those with lower household income, were also more likely to utilize a food bank, in line with data from Food Banks Canada examining community-based food services usage more generally [59]. However, our data expands on current evidence, also finding that higher food bank utilization was reported among people experiencing financial concerns, living with pre-existing mental health conditions and/or disability, who report LGBT2Q+ identity, and who had tested positive for COVID-19. Our work also expands on the data from Food Banks Canada in finding that many of these associations persisted even after controlling for household income.

These food bank usage characteristics align with prior research identifying that people who use food banks report more protracted experiences of severe poverty, financial vulnerability, food insecurity, precarious employment, and medical challenges [78–82]. An analysis of 25 years’ worth of food bank usage data analyzed shortly before the COVID-19 pandemic from a large Canadian food bank organization found that most users engage for a relatively short time, making only a few visits. Longer term and ongoing use were more likely among persons with health and mobility challenges, those reliant on disability-related income, and those supporting larger household sizes and children [80]*.* Furthermore, while most food bank users report more severe and protracted forms of food insecurity, there is little evidence that longer duration or higher frequency of food bank usage substantively diminishes the severity or stress related to food insecurity for most users [54, 83–85]. Given the cross-sectional nature of the current study design, we cannot draw inferences about whether or how access to or use of community-based food programs impacted respondents’ experiences of food worry or vice versa. However, we note that the majority (74%) of participants who reported using a food bank during the pandemic also reported sometimes or often worrying that food would run out in the previous 12 months in survey round 4.

The findings from our nationally representative data are similar to those from other countries where large unmet gaps in food needs are concentrated among people who are marginalized and/or living in poverty. Inadequate social safety nets have been documented in several other high-income countries, with growing evidence that despite the proliferation and entrenchment of food banks as a primary response to unmet food need, charity-based food approaches have failed to address the growing challenges of food insecurity before or during the COVID-19 pandemic [30, 31, 33, 57–59]. For example, Dekkinga et al. describe how food banks have become an entrenched part of the Dutch food system, while additional approaches are needed to address the root causes of food insecurity, inequality, and poverty [50]. Similarly, Barker and Russell [53] identified how the pandemic has made clear that UK food policy is overly dependent on the voluntary sector and philanthropy, including food banks. Consequently, our findings align with previous evidence that Canadian social protections failed to ensure a stable and accessible source of food for many Canadians during the pandemic, just as they had failed to do so prior to the pandemic [29]. Moreover, despite unprecedented federal investments in charitable food organizations, these findings suggest that such programs did not reach the majority of Canadians reporting food-related worries in Canada.

### Strengths and limitations

The sampling plan to facilitate this large sample size was designed to be nationally representative of the Canadian population by age, gender, region, and income [65] and facilitated participation by diverse respondents. While oversampling strategies and community partnerships were used by Maru/Matchbox to minimize selection bias and reduce technology barriers, it is possible that survey respondents differed from non-participants on other characteristics, such as ethno-racial identity and access to internet. Furthermore, as the sampling plan was not designed to over-sample for food bank users, and in light of the low rate of food bank utilization observed in our data, there were only a limited number of food bank users in the sample from which to draw conclusions. While this may limit statistical power or generalizability, our findings concerning the demographic characteristics of food bank users align with results from other population studies, including from Food Banks Canada [59].

Findings regarding the persistently high levels of food worry across survey rounds were robust to the use of two different measures of food-related worry. The first food worry item used in all four rounds of the survey was initially developed and used for pandemic research in the UK [64], to facilitate cross-national comparisons. To assess the prevalence of worrying about food due to income constraints in the Canadian context, we added a question from the Canadian Household Food Security Survey Module (HFSSM) to the second and subsequent survey rounds. However, this single item measure cannot be used as a proxy for the more fulsome 18-item validated household food security scale, which also probes experiences related to both the quantity and quality of food consumed. Therefore, the single item measures used in this study cannot be used to make direct comparisons with food insecurity data from the complete HFSSM module or from previous studies that have used different food worry-related survey items. As such, we also cannot draw conclusions about the patterns, severity or consistency of respondents’ experiences of food worry or whether respondents who drew on community food programs experienced more severe forms of food insecurity, as is reported in previous literature. Given the rapidly rising food prices due inflation in Canada in 2022 and unprecedentedly high rates of household food insecurity recorded on the national Canadian Income Survey collected in 2022 [29], it is also likely that our 2021 food worry data underestimate the income- and cost-related food worry experienced by Canadians in the months following round 4 of data collection. More recent data suggest that in 2022, both rates of household food insecurity [29] and demand for community-based food programs including food banks have risen [59]; and hence the need for continued monitoring and critical examination of these topics will continue beyond the timing of the COVID-19 pandemic context.

Other important limitations of this study’s design include the quantitative, cross-sectional nature from which the nuanced experiences of those experiencing food worry cannot be discerned. We are also unable to draw any specific conclusions here about how specific COVID-19 related policy changes or food program challenges buffered or exacerbated experiences of food worry or food program usage. For example, in the early months of the pandemic, strict physical distancing measures, and evolving public health recommendations likely shaped decisions to visit crowded spaces like food banks. Some Canadians (who were already employed or self-employed) were also able to access $500 per week income supplements for up to 28 weeks from the Canadian Emergency Response Benefit (CERB) if they were unable to work because of the pandemic [86]. While these payments have been credited with reducing the impacts of the pandemic on food security [59], high rates of food insecurity were still recorded among CERB applicants and other workers whose jobs were left precarious due to the pandemic [28]. Finally, we note that our two measures of food worry made use of different reference time frames, which are in turn different from the reference time frame queried for the food program service use item, which limits the ability to make direct comparisons between these variables.

## Conclusions

This paper provides timely insights regarding the prevalence of food-related worry and the socio-demographic characteristics of adults who accessed food banks in Canada in the context of the COVID-19 pandemic. Despite persistently high rates of food insecurity measured before and during the COVID-19 pandemic in Canada and the high rates of food-related worry reported here, relatively few adults accessed food banks or other charity-based community programs in 2020–2021. Adults facing social and structural barriers were more likely to report using a food bank during the pandemic, even after controlling for household income in statistical models, yet most food bank users still reported worry that food would run out before they got money to buy more.

Findings align with previous research, which find that Canadian policies and programs during the pandemic were insufficient to buffer against food-related worry, particularly among those already experiencing multiple dimensions of structural inequities [24, 25]. Our findings support ongoing calls to enact social policies that improve the financial circumstance of households with the lowest levels of income while also improving policies that effectively reduce the rates of food-related worry and the root causes of food insecurity more broadly [87, 88].

### Supplementary Information

**Additional file 1.**

**Additional file 2.**

## Data Availability

Data are available upon reasonable request from the corresponding author.

## Abbreviations

CMHA: Canadian Mental Health Association
HFSSM: Household Food Security Survey Module
