# Supplementary material for: Food-related worry and food bank use during the COVID-19 pandemic in Canada: results from a nationally representative multi-round study
Source: BMC Public Health. 2023 Sep 5;23:1723. doi: 10.1186/s12889-023-16602-x (PMC10478349; doi:10.1186/s12889-023-16602-x)
Supplement: Supplementary file 1 — Additional file 1. [file 12889_2023_16602_MOESM1_ESM.pdf]

maru/matchbox

Assessing the mental health impacts of COVID-19: A national survey study Introduction and Privacy Information Page
Final Questionnaire: Click or tap to enter a date. Version: 1.0

SURVEY DETAILS

Survey Respondent Details:

Quote Code: Add quote code
Languages: English

Device Type: Desktop + Mobile
Target LOI: Add length in minutes

Standard Demographics:

Below are the standard demographics that are automatically included in projects using Maru/Blue sample. Click on each demographic to preview the question text, answer options and standard rollups where available:

Age *(incl. Age Rollup)*
Gender
Regions
- CDA: Postal Code, Province, SAC
- US: Zipcode, Division, 4 Regions, DMA, FIPS
- UK: Post Code, 14 Region Rollup

Education
Employment
Marital Status
Ethnicity (incl. Hispanic/Latino in US)
Household Income
Children in Household

Below are the data analysis requirements pertaining to the standard demographic questions above *(i.e. if an age roll-up with different breaks than the standard is required, please outline as below):*

QUESTION	REQUIREMENTS
**Age**	18-24 years, 25-34 years, 35-44 years, 45-54 years, 55-64 years, 65-74 years, 75+
**Choose a demo**	
**Choose a demo**	

Key Questions:

Below are the key/core questions that are critical to this research and will be the focus of reporting and analysis:

- Q#
- Q#
- Q#

Study Timelines:

Below are the study milestones and associated timelines:

MILESTONE	DATE
**Draft Questionnaire Delivered:**	Click or tap to enter a date.
**Final Questionnaire Approved:**	Click or tap to enter a date.
**Programming + QA:**	Click or tap to enter a date. **to** Click or tap to enter a date.
**Fielding:**	Click or tap to enter a date. **to** Click or tap to enter a date.

Deliverables:	Click or tap to enter a date.

Survey Quotas:

Below are the entry and in-study quota details:

QUOTA	CRITERIA	AMOUNT	HARD / SOFT	MIN / MAX
Entry Quota	Total Respondents			

Termination Text:

If your study is using IC or Client Supplied sample, please specify termination text below or delete this section if not applicable.

TERMINATION TYPE	TEXT	REDIRECT (if applicable)
Disqualification		
Over Quota		
Complete		

SECTION 1: PREAMBLE AND CONSENT

maru/matchbox

[QUESTION TYPE: single choice LAYOUT: default (kato)]
[QCONSENT] [REQUIRED]
Base: Total

Thank you for your interest in the COVID-19 Mental Health Survey Study. Completing this anonymous survey provides us with information that can help community organizations, and public health leaders understand and respond to the mental health impacts of the pandemic. The information you give will be kept private and confidential.

The survey will take about 10 minutes to complete.

If you agree to take part in the survey, you will be asked about your feelings, attitudes, mental and general health.

This survey is voluntary and you may withdraw at any time by closing the survey window. This will exit the survey and delete all responses you have entered. After clicking the "submit survey" button at the end of the survey, you will be unable to withdraw your responses.

If you would like to participate in the survey, please read the following privacy statement and complete the consent section below.

Privacy Statement

This information is collected by the COVID-19 Mental Health Research Team, led by Dr. Emily Jenkins at the University of British Columbia. Survey data will be used to understand the mental health impacts of COVID-19 on those living in Canada during the study period.

Should you have any questions, or would like more information, please contact:
Dr. Emily Jenkins (Principal Investigator) 604-822-4980 or emily.jenkins@ubc.ca

Only the research team will have access to the survey responses; the anonymized responses will be pooled and stored in a password protected database on a Canadian-hosted server. All information will remain in Canada. Electronic files will be password protected and erased using appropriate file deletion software. The findings of the research may be published in open access journals, which may allow other researchers to access the anonymous survey responses. Your name will never be associated with the data and will not appear in any publications.

Ethics Review

If you have any concerns or complaints about your rights as a research participant and/or your experiences while participating in this study, contact the Research Participant Complaint Line in the UBC Office of Research Ethics at 604-822-8598 or if long distance e-mail RSIL@ors.ubc.ca or call toll free 1-877-822-8598.

Checking the box below indicates that you have read and understand the above information.

[ANSWERS] [ANCHOR]
I have read and agree to the terms of the above Privacy Statement, and consent to participate in this survey.
I do not wish to continue **[TERMINATE]**

TAB NOTE:
Detail Table Requirements

maru/matchbox

[QUESTION TYPE: text instruction]
[Q_INSTRUCTION] [REQUIRED]
Base: Total

The 2019 novel coronavirus (2019-nCoV), otherwise known as COVID-19, is an infectious disease that has resulted in a global pandemic. Throughout this questionnaire, we will refer to the disease as COVID-19.

For the following questions, please think about yourself, members of your household, or other family members who have been affected by the COVID-19 pandemic.

[QUESTION TYPE: open end LAYOUT: text field (single line) narrowtextbox - numeric (kato)]
[QS2] [REQUIRED]
Base: Total

What's your age?

[TERMINATE IF NOT 18+ & Show numbers in drop down 1 to 100]

TAB NOTE:

Breakdown: 18-24 years, 25-34 years, 35-44 years, 45-54 years, 55-64 years, 65-74 years, 75+

[QUESTION TYPE: single choice LAYOUT: default (kato)]
[QS3] [REQUIRED]
Base: Total

In which province or territory do you currently live?

[ANSWERS] [ANCHOR]
Alberta
British Columbia
Manitoba
New Brunswick
Newfoundland and Labrador
Northwest Territories
Nova Scotia
Nunavut
Ontario
Prince Edward Island
Quebec
Saskatchewan
Yukon

TAB NOTE:
Detail Table Requirements

[QUESTION TYPE: single choice LAYOUT: default (kato)]
[QS4] [REQUIRED]
Base: Total

Do you live in an urban or rural environment?

[ANSWERS] [ANCHOR]
Urban
Suburban
Rural

TAB NOTE:
Detail Table Requirements

[QUESTION TYPE: single choice **LAYOUT:** default (kato)**]**
[QS5] [REQUIRED]
Base: Total

Please select the highest level of education you have completed.

[ANSWERS] [ANCHOR]
Elementary/grade school
Some high school
High school graduate
Some college / technical school
Completed college / technical school
Some university
University undergraduate degree
Some post-graduate school
Post-graduate degree

TAB NOTE:
Detail Table Requirements

[QUESTION TYPE: single choice **LAYOUT:** default (kato)**]**
[QS6] [REQUIRED]
Base: Total

What is your marital status?

[ANSWERS] [ANCHOR]
Single, never married
Common law
Civil partnership
Married
Separated
Divorced
Widowed
Engaged

TAB NOTE:
Detail Table Requirements

maru/matchbox

[QUESTION TYPE: single choice LAYOUT: default (kato)]
[QS7] [REQUIRED]
Base: Total

Please indicate the category which best describes your total household annual income before taxes.

[ANSWERS] [ANCHOR]
Less than $25,000
$25,000 to less than $35,000
$35,000 to less than $50,000
$50,000 to less than $75,000
$75,000 to less than $100,000
$100,000 to less than $125,000
$125,000 to less than $150,000
$150,000 to less than $250,000
$250,000 to less than $500,000
$500,000 or more
Don’t know/prefer not to say

TAB NOTE:
Detail Table Requirements

SECTION 2: COVID QUESTIONS

[QUESTION TYPE: multi choice LAYOUT: default (kato)]
[Q_EMPLOYMENTSTATUS] [REQUIRED]
Base: Total

The COVID-19 pandemic has had substantial impacts on employment. Which of the following best describes your current employment status? (Please select all that apply)

[ANSWERS] [ANCHOR]
Working full time (30 or more hours per week)
Working part time (fewer than 30 hours per week)
Full time student (e.g. school, college, university, job training)
Part time student (e.g. school, college, university, job training)
Not working (e.g. parental leave, disability, medical leave, etc.)
Not working due to personal preference because of COVID-19
Volunteer (unpaid)
Retired
Unemployed (due to COVID-19)
Unemployed (prior to COVID-19)
Underemployed (loss of hours due to COVID)

Other
Prefer not to answer **[EXCLUSIVE]**

TAB NOTE:
Detail Table Requirements

[QUESTION TYPE: single choice **LAYOUT:** default (kato)**]**
[Q_ESSENTIALSERVICE] [REQUIRED]
Base: IF CODE 1-2 in Q_EMPLOYMENTSTATUS

The job that I am currently working in has been deemed as an essential service during the COVID-19 pandemic (i.e., your position is identified by your provincial government as an essential service and you have continued to work in your role throughout the pandemic).

[ANSWERS] [ANCHOR]
Yes
No
Prefer not to answer

TAB NOTE:
Detail Table Requirements

[QUESTION TYPE: single choice **LAYOUT:** default (kato)**]**
[Q_EMPLOYMENTTYPE] [REQUIRED]
Base: [CODE 1-2 EMPLOYMENT STATUS (working full or part time)]

Please select the category that **BEST** describes your professional role:

[ANSWERS] [RANDOMIZE]
Health and health services
Law enforcement, public safety, first responder
Vulnerable population service provider (e.g., community outreach, substance use and addiction services)
Education (K-12) **[GROUP EDUCATION]**
Education (post-secondary) **[GROUP EDUCATION]**
Education (early childhood) **[GROUP EDUCATION]**
Food and agriculture service provider (farming, food processing, grocery, hardware)
Transportation
Industry and manufacturing
Communications and information technology
Financial institutions
Retail
Other **[ANCHOR]**
Prefer not to answer **[ANCHOR]**

TAB NOTE:
Detail Table Requirements

[QUESTION TYPE: single choice LAYOUT: default (kato)]
[QSelfMentalhealth] [REQUIRED]
Base: Total

In general, would you say your mental health is:

[ANSWERS] [ANCHOR]
Excellent
Very good
Good
Fair
Poor

TAB NOTE:
Detail Table Requirements

[QUESTION TYPE: single choice LAYOUT: default (kato)]
[Q_Mentalhealth] [REQUIRED]
Base: Total

Compared to before the COVID-19 pandemic and related restrictions in Canada, how would you say your mental health is **now**?

[ANSWERS] [ANCHOR]
Significantly better now
Slightly better now
About the same
Slightly worse now
Significantly worse now
Prefer not to answer

TAB NOTE:
Detail Table Requirements

[QUESTION TYPE: text instruction]

[Q_K6] [REQUIRED] [STRAIGHTLINE CHECK: NO]
Base: Total

The following questions ask about how you have been feeling during the past 30 days. For each question, please indicate how often you have this feeling.

[QUESTION TYPE: single choice LAYOUT: default (kato)]

maru/matchbox

[Q1a] [REQUIRED]
Base: Total

During the past 30 days, how often did you feel **nervous**?

[ANSWERS] [ANCHOR]
All of the time
Most of the time
Some of the time
A little of the time
None of the time

TAB NOTE:
Detail Table Requirements

[QUESTION TYPE: single choice **LAYOUT: default (kato)]**
[Q1b] [REQUIRED]
Base: Total

During the past 30 days, how often did you feel **hopeless**?

[ANSWERS] [ANCHOR]
All of the time
Most of the time
Some of the time
A little of the time
None of the time

[QUESTION TYPE: single choice **LAYOUT: default (kato)]**
[Q1c] [REQUIRED]
Base: Total

During the past 30 days, how often did you feel **restless or fidgety**?

[ANSWERS] [ANCHOR]
All of the time
Most of the time
Some of the time
A little of the time
None of the time

[QUESTION TYPE: single choice **LAYOUT: default (kato)]**
[Q1d] [REQUIRED]
Base: Total

maru/matchbox

During the past 30 days, how often did you feel **so depressed that nothing could cheer you up**?

[ANSWERS] [ANCHOR]
All of the time
Most of the time
Some of the time
A little of the time
None of the time

[QUESTION TYPE: single choice **LAYOUT: default (kato)]**
[Q1e] [REQUIRED]
Base: Total

During the past 30 days, how often did you feel **that everything was an effort**?

[ANSWERS] [ANCHOR]
All of the time
Most of the time
Some of the time
A little of the time
None of the time

[QUESTION TYPE: single choice **LAYOUT: default (kato)]**
[Q1f] [REQUIRED]
Base: Total

During the past 30 days, how often did you feel **worthless**?

[ANSWERS] [ANCHOR]
All of the time
Most of the time
Some of the time
A little of the time
None of the time

[QUESTION TYPE: multi choice **LAYOUT:** default (kato)**]**
[Q_COVID19disease] [REQUIRED]
Base: Total

Which of the following applies to how you have been affected by COVID-19 at any point during the pandemic? (Please select all that apply)

[ANSWERS] [ANCHOR]
I have been tested for COVID-19 and had a positive result
I have been tested for COVID-19 for employment/education reasons and had a negative result
I have been tested for COVID-19 for travel reasons and had a negative result
I have been tested for COVID-19 because I had symptoms and/or contact with someone who tested positive, and

had a negative result
I had symptoms of COVID-19, but was not able to get tested
Someone else in my household has tested positive for COVID-19
I am living with residual symptoms (“Long COVID”) after having contracted COVID-19
I am caring for someone with residual symptoms (“Long COVID”) after having contracted COVID-19
A close friend or family member has died from COVID-19
A close friend or family member has been hospitalized due to COVID-19
I have been hospitalized due to COVID-19
None of these **[ANCHOR] [EXCLUSIVE]**
Don’t know **[ANCHOR] [EXCLUSIVE]**
Prefer not to answer **[ANCHOR] [EXCLUSIVE]**

TAB NOTE:
Detail Table Requirements

[QUESTION TYPE: multi choice **LAYOUT:** default (kato)**]**
[Q_EMOTIONALRESPONSE] [REQUIRED]
Base: Total

Which of the following emotions have you felt as a result of the COVID-19 pandemic in the **past 2 weeks**?
(Please select all that apply)

[ANSWERS] [RANDOMIZE]
Afraid
Panicked
Anxious or worried
Empathetic
Indifferent
Hopeful
Hopeless
Ashamed
Guilty
Lonely or isolated
Unprepared
Sad
Angry
Stressed
Bored
Inspired
Depressed
Calm
Comfortable
Content
Secure
None of these **[ANCHOR] [EXCLUSIVE]**
Don’t know **[ANCHOR] [EXCLUSIVE]**
Prefer not to answer **[ANCHOR] [EXCLUSIVE]**

maru/matchbox

TAB NOTE:
Detail Table Requirements

[QUESTION TYPE: single choice grid **LAYOUT:** default (kato)**]**
[Q_STRESSORS] [REQUIRED]
Base: Total

Have you been stressed or worried about any of the following as a result of the COVID-19 pandemic in **the past 2 weeks?** (Please select one option on each row)

[COLUMNS] [ANCHOR]
Yes
No
Don't know /Not applicable/ Prefer not to say

[ROWS] [RANDOMIZE]
Financial concerns (e.g. going into debt, ability to pay bills, long-term economic impacts, etc.)
Being unable to access government benefit payments (e.g., not being eligible for benefits)
Losing benefits due to reaching benefit limit
Being in close proximity to unvaccinated family, colleagues or peers
Losing my job / loss of my job
Changing work requirements (e.g., return to in-person work environment)
Having to leave my job due to stress, fatigue, and/or burnout
Being able to cope with uncertainty (e.g. not knowing what will happen)
Becoming ill with the virus
Having no-one to care for me as a result of becoming ill with the virus
Not being able to care for friends and family as a result of becoming ill with the virus
The spread of new variants/mutations of the virus
Being vulnerable because other people are not following public health orders
Being vulnerable because of an existing medical condition, age, etc.
Being separated from friends and family
Having enough food to meet my household's basic needs
My education or career training being interrupted
Making my existing mental health problems worse
Worrying about how the mental health of my child(ren) will be affected by the pandemic
My child contracting COVID-19 at childcare/daycare/school
My child bringing the virus into the household and infecting other people
Public health measures negatively impacting my child's ability to learn (e.g., distancing, masking)
Potential for my child's school to close due to COVID-19 outbreak
Reopening of my workplace
Physical distancing measures negatively impacting my child's ability to play and connect with friends
Contracting the virus in my work setting
The safety or effectiveness of COVID-19 vaccines
The duration of protection provided by the vaccine(s)

The safety of a COVID-19 vaccine for children under 12 years of age
The effectiveness of a COVID-19 vaccine for children under 12 years of age
My child being unvaccinated
Worrying about COVID-19 becoming endemic (meaning it will continue to circulate among pockets of the population for years to come)
Lost connections with family and friends
Worrying about future lockdowns
Worrying about the long term mental health impacts resulting from the stress of the pandemic
Experiencing relationship challenges with my partner
Being safe from physical or emotional domestic violence
Fear of getting severely sick or dying
Fear of a family member/loved one getting severely sick or dying
Challenges in accessing what I need as a result of supply chain disruptions
Worrying about the compounding effects of COVID-19 alongside the climate crisis
Utilizing 911 or emergency response services for a mental health crisis

TAB NOTE:
Detail Table Requirements

[QUESTION TYPE: single choice **LAYOUT:** default (kato)**]**
[QFOODSECURITY_A] [REQUIRED]
Base: Total

You and other household members worried that food would run out before you got money to buy more. Was that often true, sometimes true, or never true in the last 12 months?

[ANSWERS] [ANCHOR]
Often true
Sometimes true
Never true
Don’t know/prefer not to answer

TAB NOTE:
Detail Table Requirements

[QUESTION TYPE: multi choice **LAYOUT:** default (kato)**]**
[Q_FOODSECURITY] [REQUIRED]
Base: Total

Since the onset of the COVID-19 pandemic and related restrictions in Canada, have you or any members of your household accessed food-based community programs to get food? (please select all that apply)

[ANSWERS] [RANDOMIZE]
Food Bank
Soup Kitchens/Free Meal programs

maru/matchbox

Meal or food programs from a school
Community Kitchen program
Community Garden
Food voucher program (e.g., receiving gift cards for food from a charitable organization)
Food delivered by a community program
Asking friends or family for help with food
Other **[ANCHOR]**
No – I haven't accessed any food programs **[EXCLUSIVE] [ANCHOR]**

TAB NOTE:
Detail Table Requirements

[QUESTION TYPE: single choice **LAYOUT:** default (kato)**]**
[Q_OVERSTRESSCOPE] [REQUIRED]
Base: Total

Overall, how well do you think you are coping with stress related to the COVID-19 pandemic?

[ANSWERS] [ANCHOR]
Very well
Fairly well
Not very well
Not well at all
Don't know
Prefer not to say
Not applicable – I have not experienced any stress related to COVID-19

TAB NOTE:
Detail Table Requirements

[QUESTION TYPE: multi choice **LAYOUT:** default (kato)**]**
[Q_COPING1] [REQUIRED]
Base: Total

Which of the following have helped you to cope with **stress** related to the COVID-19 pandemic in the **past 2 weeks**? (Please select all that apply)

[ANSWERS] [RANDOMIZE]
Connecting with those in my household
Connecting with my family or friends **virtually** (e.g., phone, video chat, etc.)
Connecting in-person with friends or family
Enjoying outdoor activities with friends or family
Connecting with a mental health worker or counsellor **virtually** (e.g. via phone, video chat, etc.)
Having a supportive employer
Spending time with my pet(s)
Receiving in-person mental health supports
Accessing virtual mental health resources (e.g. online cognitive behavioural therapy, etc.)

Maintaining a healthy lifestyle (e.g. balanced diet, enough sleep, exercise, etc.)
Keeping up to date with relevant information (e.g. TV news, newspapers, online information, etc.)
Limiting my exposure to the news about COVID-19
Limiting exposure to social media (e.g. Facebook, Instagram, Snapchat, Twitter etc.)
Increasing my use of social media (e.g. Facebook, Instagram, Snapchat, Twitter etc.)
Contacting a support group (i.e., where members with the same issues can come together for sharing coping strategies, to feel more empowered and/or for a sense of community)
Going for a walk/exercise outside
Exercising indoors (e.g., gym, home)
Doing a hobby
Learning or doing something new
Volunteering to help
Accessing federal government benefits and supports (e.g., Employment Insurance, Canada Recovery Benefit, Canada Recovery Sickness Benefit, etc.)
Accessing provincial government supports (e.g., emergency benefits for workers, rent freezes)
Other [open] please specify [anchor]
Don't know **[ANCHOR] [EXCLUSIVE]**
Nothing has helped me to cope with my stress related to COVID-19 **[ANCHOR] [EXCLUSIVE]**
Not applicable – I don't feel stressed **[ANCHOR] [EXCLUSIVE]**

TAB NOTE:
Detail Table Requirements

[QUESTION TYPE: single choice **LAYOUT:** default (kato)**]**
[QVIRTUAL] [REQUIRED]
Base: Total

If you have experienced mental health challenges at any point during the pandemic, have you used virtual (online or phone-based) mental health services and supports (e.g., counselling, online cognitive behavioural therapy, mental health coaching sessions)?

[ANSWERS] [ANCHOR]
Yes
No
Not applicable. I haven't experienced a mental health challenge during the pandemic
Prefer not to say

TAB NOTE:
Detail Table Requirements

[ASK QVIRTUAL_2, IF NO IN QVIRTUAL]
[QUESTION TYPE: multi choice **LAYOUT:** default (kato)**]**
[QVIRTUAL2] [REQUIRED]
Base: If No (Code 2) in QVirtual

Virtual mental health supports are receiving substantial investment to help people cope with mental health challenges during the pandemic. In order to inform better programs, could you please indicate why you did not access virtual mental health supports. (Select all that apply)

[ANSWERS] [RANDOMIZE]
Stigma (I was afraid of what others would think of me)
Didn't feel I needed help
Privacy concerns
I don't think they would be helpful
I didn't know these supports were available
Necessary equipment not available (e.g., computer, smart phone)
Connectivity issues (e.g., no/slow internet connection)
Competing demands on my time
I prefer in-person health care supports
Other (Please specify) **[ANCHOR]**

TAB NOTE:
Detail Table Requirements

[QUESTION TYPE: single choice grid **LAYOUT:** default (kato)**]**
[QVIRTUAL3] [REQUIRED]
Base: Total

Are you aware of any of the following virtual mental health supports in Canada?

[COLUMNS] [ANCHOR]
Yes
No
Don't know /Not applicable/ Prefer not to say

[ROWS] [RANDOMIZE]
BounceBack
Wellness Together Canada
Strongest Families
WellCan
MindBeacon
Other **[ANCHOR]**

TAB NOTE:
Detail Table Requirements

[QUESTION TYPE: single choice **LAYOUT:** default (kato)**]**
[QHelp] [REQUIRED]
Base: Total

Since the start of the COVID pandemic), was there ever a time when you felt that you needed help for problems with your emotions, mental health or use of alcohol or drugs, but you didn't receive it?

maru/matchbox

[ANSWERS] [ANCHOR]
Yes
No

TAB NOTE:
Detail Table Requirements

[QUESTION TYPE: multi choice **LAYOUT:** default (kato)**]**
[QHelp_1] **[REQUIRED]**
Base: Those who selected 'Yes' in QHelp

Why didn't you receive the help you needed (during the past 12 months)? (Select all that apply)

[ANSWERS] [ANCHOR]
You preferred to manage yourself
You didn't know how or where to get this kind of help
You haven't gotten around to it (e.g., too busy)
Your job interfered (e.g., workload, hours of work or no cooperation from supervisor)
Access to care was limited (e.g., help was not readily available)
You didn't have confidence in the health care system or social services
You couldn't afford to pay
Insurance did not cover
You were afraid of what others would think of you
Language problems
Talked to friends or family instead
Hoped your mental health would get better by itself
Other **[ANCHOR]**

TAB NOTE:
Detail Table Requirements

[QUESTION TYPE: multi choice grid **LAYOUT:** default (kato)**]**
[Q_COPING2] **[REQUIRED]**
Base: Total

Please indicate how your use of any of the following has been impacted by the COVID-19 pandemic? (Please select one option on each row)
[COLUMNS] [ANCHOR]
More
Less
No change
Not applicable
Prefer not to say

[ROWS] [RANDOMIZE]

maru/matchbox

Drinking alcohol
Use of tobacco products (e.g. cigarettes, cigars, chewing tobacco, vaping, etc.)
Use of cannabis products
Use of prescribed medication
Use of other psychoactive substances (e.g., cocaine, heroin)
Gambling
Eating/Food consumption
Screen time
Compulsive online shopping (e.g. buying things you don’t really need)

TAB NOTE:
Detail Table Requirements

[QUESTION TYPE: single choice **LAYOUT:** default (kato)**]**
[QCOPING3] [REQUIRED]
Base: Total

Has your use of substances increased as a way to cope at any point during the pandemic?

[ANSWERS] [ANCHOR]
Yes
No
Prefer not to say

TAB NOTE:
Detail Table Requirements

[QUESTION TYPE: single choice **LAYOUT:** default (kato)**]**
[QUS_AuditC_1] [REQUIRED]
Base: Total

Think about your drinking in the past year. A drink means one beer, one small glass of wine (5oz) or one mixed drink containing one shot (1.5oz) of spirits.

How often do you have a drink containing alcohol?

[ANSWERS] [ANCHOR]
Never
Less than Monthly
Monthly
Weekly
2-3 times a Week
4-6 times a Week
Daily

TAB NOTE:

maru/matchbox

Detail Table Requirements

[QUESTION TYPE: single choice LAYOUT: default (kato)]
[QUS_AuditC_2] [REQUIRED]
Base: Total

How many drinks containing alcohol do you have on a typical day you are drinking?

[ANSWERS] [ANCHOR]
1 Drink
2 Drinks
3 Drinks
4 Drinks
5-6 Drinks
7- 9 Drinks
10 or more drinks

TAB NOTE:
Detail Table Requirements

[QUESTION TYPE: single choice LAYOUT: default (kato)]
[QUS_AuditC_3] [REQUIRED]
Base: Total

How often do you have X (5 for men; 4 for women and men over age 65) or more drinks on one occasion?

[ANSWERS] [ANCHOR]
Never
Less than Monthly
Monthly
Weekly
2-3 times a Week
4-6 times a Week
Daily

TAB NOTE:
Detail Table Requirements

SECTION 3: Self-harm section

[QUESTION TYPE: text instruction**]**
[Q_S3INSTRUCTION] [REQUIRED]
Base: Total

The following questions are on the topic of self-harm and suicidal thoughts. We understand this can be a sensitive topic, so please remember that your answers are anonymous. If you are in crisis, please call 1-833-456-4566 toll

free (In QC: 1-866-277-3553), 24/7 or visit www.crisisservicescanada.ca

[QUESTION TYPE: single choice grid **LAYOUT:** default (kato)**]**
[Q_selfharm1] [REQUIRED]
Base: Total

Have you done or experienced any of the following, as a result of the COVID-19 pandemic in the **past 2 weeks**? (Please select one option on each row)

[COLUMNS] [ANCHOR]
Yes
No
Prefer not to say

[ROWS] [RANDOMIZE]
Experienced suicidal thoughts/feelings
Deliberately hurt myself
TAB NOTE:
Detail Table Requirements

[ONLY SHOW IF SELECTED 'YES TO ANY RESPONSE IN Q SELFHARM1]
[QUESTION TYPE: single choice grid **LAYOUT:** default (kato)**]**
[Q_selfharm2] [REQUIRED]
Base: IF YES TO ANY ABOVE

How often have you experienced or done each of the following as a result of the COVID-19 pandemic in the **past 2 weeks**? (Please select one option on each row)

[COLUMNS] [ANCHOR]
Once a day or more often
Nearly everyday day
A few times a week
Passing thoughts
Don't know
Prefer not to say

[ROWS] [ANCHOR]
Experienced suicidal thoughts/feelings
Deliberately hurt myself

TAB NOTE:
Detail Table Requirements

SECTION 4: DEMOGRAPHICS

maru/matchbox

[QUESTION TYPE: single choice **LAYOUT:** default (kato)**]**
[QSEX] [REQUIRED]
Base: Total

What sex where you assigned at birth?

[ANSWERS] [ANCHOR]
Male
Female

TAB NOTE:
Detail Table Requirements

[QUESTION TYPE: single choice **LAYOUT:** default (kato)**]**
[Q_GENDER] [REQUIRED]
Base: Total

We know that gender has important consequences for health and how we are treated by different individuals and institutions. Which gender do you most identify with?

[ANSWERS] [ANCHOR]
Woman
Man
Non-binary
Two-Spirit
Not listed
Prefer not to answer

TAB NOTE:
Detail Table Requirements

[QUESTION TYPE: multi choice **LAYOUT:** default (kato)**]**
[Q_ETHNICITY] [REQUIRED]
Base: Total

What is your ethnic origin? Ethnic origin refers to the ethnic or cultural origins of your ancestors (who are usually more distant than a grandparent) (Check all that apply)

[ANSWERS] [RANDOMIZE]
Indigenous origins (for example, First Nations, Inuit, Métis)
East Asian origins (for example, Chinese, Japanese, Korean)
South Asian origins (for example, Indian, Punjabi, Pakistani)
Southeast Asian origins (for example, Filipino, Thai, Vietnamese)
Latin American origins (for example, Brazilian, Cuban, Bolivian)
European origins (for example, British, German, Russian)

maru/matchbox

Middle Eastern origins (for example, Iranian, Iraqi, Afghan)
African origins (for example, Nigerian, Ghanaian, Zimbabwean)
Other (please specify) ______________________________ **[ANCHOR]**
Don't know **[EXCLUSIVE] [ANCHOR]**
Prefer not to answer **[EXCLUSIVE] [ANCHOR]**

TAB NOTE:
Detail Table Requirements

[QUESTION TYPE: multi choice **LAYOUT:** default (kato)**]**
[QRACE] [REQUIRED]
Base: Total

We know that people of different races do not have significantly different genetics. But our race still has important consequences, including how we are treated by different individuals and institutions. Which race category best describes you? Check all that apply:

[ANSWERS] [ANCHOR]
Black (African, Afro-Caribbean, African Canadian descent)
East Asian (Chinese, Korean, Japanese, Taiwanese descent)
Southeast Asian (Vietnamese, Cambodian, Thai, Filipino, Indonesian, other Southeast Asian descent)
Indigenous (First Nations, Métis, Inuk/Inuit descent)
Latino (Latin American, Hispanic descent)
Middle Eastern (Arab, Persian, West Asian descent (e.g. Afghan, Egyptian, Iranian, Lebanese, Turkish, Kurdish)
South Asian (East Indian, Pakistani, Bangladeshi, Sri Lankan, Indo-Carribean descent)
White (European descent)
Another race category (Includes values not described above)
Do not know
Prefer not to answer

TAB NOTE:
Detail Table Requirements

[QUESTION TYPE: single choice **LAYOUT:** default (kato)**]**
[Q_SEXUALITY] [REQUIRED]
Base: Total

Do you identify as being LGBT2Q+ (lesbian, gay, bisexual, trans, Two-Spirit, queer, etc.)?

[ANSWERS] [ANCHOR]
Yes
No
Unsure
Prefer not to answer

TAB NOTE:

maru/matchbox

Detail Table Requirements

[QUESTION TYPE: single choice **LAYOUT:** default (kato)**]**
[Q_DISABILITY] [REQUIRED]
Base: Total

Do you identify as a person with a disability?

[ANSWERS] [ANCHOR]
Yes
No
Prefer not to answer

TAB NOTE:
Detail Table Requirements

[QUESTION TYPE: single choice **LAYOUT:** default (kato)**]**
[QMentalHealth_Pre] [REQUIRED]
Base: Total

Do you identify as a person who has a pre-existing (prior to COVID-19) mental health condition?

[ANSWERS] [ANCHOR]
Yes
No
Prefer not to answer

[QUESTION TYPE: multi choice **LAYOUT:** default (kato)**]**
[QMentalHealth_subtype] [REQUIRED]
BASE: answered yes to QMentalHealth_Pre

The COVID-19 pandemic is impacting people with mental health conditions in various ways. To help us gain a better understanding of this, please identify the mental health condition that you experience (check all that apply).

[ANSWERS] [ANCHOR]

Mood disorder (e.g., depression, bipolar disorder)
Anxiety disorder
Personality disorder
Psychotic disorder (e.g., schizophrenia)
Eating disorder
Trauma-related disorder (e.g., post-traumatic stress disorder)
Substance use disorder
Other **[ANCHOR]**

Prefer not to answer **[Exclusive] [Anchor]**

TAB NOTE:
Detail Table Requirements
[QUESTION TYPE: single choice **LAYOUT:** default (kato)**]**
[Q_CITIZENSHIP] [REQUIRED]
Base: Total

Which of the following best describes your Canadian citizenship status?

[ANSWERS] [ANCHOR]
Canadian citizen by birth
Canadian citizen by naturalization
Landed immigrant/Permanent resident
Refugee
Not a citizen
Prefer not to say

TAB NOTE:
Detail Table Requirements

[QUESTION TYPE: single choice **LAYOUT:** default (kato)**]**
[Q_VACCINATION] [REQUIRED]
Base: Total

We are keen to hear from people who are both vaccinated and unvaccinated as some research indicates that mental health outcomes differ by vaccine status. Are you considered fully vaccinated against COVID-19 (i.e., received the full series of an accepted (endorsed by the World Health Organization) COVID-19 vaccine or a full series of a combination of accepted vaccines at least 14 full days prior to completing this survey).

[ANSWERS] [ANCHOR]
Yes
No
Prefer not to answer

[QUESTION TYPE: single choice **LAYOUT:** default (kato)**]**
[Q_PARENT2] [REQUIRED]
Base: Total

How many children (under 18 years of age) reside in your household?

[ANSWERS] [ANCHOR]
0
1
2
3+

maru/matchbox

TAB NOTE:
Detail Table Requirements

[QUESTION TYPE: single choice **LAYOUT:** default (kato)**]**
[Q_ParentGuardianStatus] [REQUIRED]
Base: Total

Which of the following best describes your parental/guardian status?

[ANSWERS] [ANCHOR]
Not a parent / guardian
Parent / guardian (to a child of any age)
Prefer not to say

TAB NOTE:
Detail Table Requirements

[QUESTION TYPE: multi choice **LAYOUT:** default (kato)**]**
[Q_CHILDAGE] [REQUIRED]
Base: if yes at Q_ParentGuardianStatus

What age group is/are your child(ren)? (Please select all that apply)

[ANSWERS] [ANCHOR]
4 years and under
5-11 years
12-17 years
18 years and over

TAB NOTE:
Detail Table Requirements

[QUESTION TYPE: single choice **LAYOUT:** default (kato)**]**
[QPARENT3] [REQUIRED]
Base: Total

Are you a single parent?

[ANSWERS] [ANCHOR]
Yes
No
Prefer not to say

TAB NOTE:
Detail Table Requirements

[QUESTION TYPE: multi choice **LAYOUT:** default (kato)**]**
[QSCHOOL] [REQUIRED]
Base: if yes at Q_ParentGuardianStatus

Which of the following best describes the childcare/daycare or school situation of your child/children?

[ANSWERS] [ANCHOR]
One (or more) of my child/ren is attending childcare/daycare
One (or more) of my child/ren is attending school
One (or more) of my child/ren are at home due to concerns about the COVID-19 pandemic
One (or more) of my children is homeschooled due to the COVID-19 pandemic
One (or more) of children is homeschooled independent of the COVID-19 pandemic
Other **[ANCHOR]**
Prefer not to say **[EXCLUSIVE] [ANCHOR]**

TAB NOTE:
Detail Table Requirements

[QUESTION TYPE: single choice **LAYOUT:** default (kato)**]**
[Q_CHILDMENTALHEALTH] [REQUIRED]
Base: if yes at Q_ParentGuardianStatus

Compared to before the COVID-19 pandemic and related restrictions in Canada, how would you say the mental health **of your child(ren)** is now?

[ANSWERS] [ANCHOR]
Significantly better now
Slightly better now
About the same
Slightly worse now
Significantly worse now
It is affecting my children differently (some feel better/some feel worse)
Prefer not to answer

[QUESTION TYPE: single choice grid **LAYOUT:** default (kato)**]**
[Q_CHILDRENMENTALHEALTH_BriefSCAS] [REQUIRED] [STRAIGHTLINE CHECK: NO]
Base: Total

Below is a list of items that describe children. For each item please circle the response that best describes your child. Please answer all the items. There is no set time period over which the judgement has to be made.

[COLUMNS] [ANCHOR]
Never
Sometimes
Often
Always

[ROWS] [ANCHOR]
My child worries that something bad will happen to him/her

maru/matchbox

My child complains of feeling afraid
All of a sudden my child feels really scared for no reason at all
My child worries about being away from us / me
My child worries what other people think of him/her
My child has trouble going to school in the mornings because (s)he feels nervous or afraid
My child feels afraid that (s)he will make a fool of him/herself in front of people
My child worries about things

TAB NOTE:
Detail Table Requirements

[QUESTION TYPE: multi choice **LAYOUT:** default (kato)**]**
[Q_ChildCopingStrategies] [REQUIRED]
Base: if yes at Q_ParentGuardianStatus

Which do you think has helped your child(ren) cope with **stress** related to COVID-19 pandemic in the **past 2 weeks**? (Please select all that apply)

[ANSWERS] [RANDOMIZE]
Connecting with family who live outside our home **virtually** (e.g. phone, video chat, text etc.)
Connecting with friends **virtually** (e.g. phone, video chat, text etc.)
Contacting a **school or community-based** mental health worker or counsellor **virtually** (e.g. via phone, video chat, etc.)
Receiving **in-person** mental health supports
Accessing virtual mental health resources through medical professionals (e.g. online cognitive behavioural therapy, etc.)
Accessing virtual educational or self-help mental health resources through websites, apps, or phone (e.g., Headspace, KidsHelpPhone)
Participating in a virtual child/youth support group
Maintaining a healthy lifestyle (e.g. balanced diet, enough sleep, exercise, etc.)
Maintaining family routines (e.g., family meals, bedtime routines)
Keeping up to date with relevant information (e.g. TV news, newspapers, online information, etc.)
Limiting their exposure to the news about COVID-19
Limiting their exposure to social media (e.g. Facebook, Instagram, Snapchat, Twitter etc.)
More time for social media use (e.g. Facebook, Instagram, Snapchat, Twitter etc.)
Going for a walk/exercise outside
Exercising in our home
Spending time with pet(s)
Playing outdoors
Playing inside (e.g., games, toys, telling stories)
Doing a hobby (e.g., music, reading, arts & crafts)
Volunteering to help
Connecting **in person** with friends
Connecting **in person** with teachers/childcare providers
Connecting **in person** with friends outside of school/childcare
Other (please specify) **[ANCHOR]**

Don't know **[ANCHOR] [EXCLUSIVE]**
Not applicable **[ANCHOR] [EXCLUSIVE]**
Nothing has helped my child(ren) to cope with stress related to COVID-19 **[ANCHOR] [EXCLUSIVE]**

TAB NOTE:
Detail Table Requirements

[QUESTION TYPE: single choice grid **LAYOUT:** default (kato)**]**
[Q_PARENT_CHILD_INTERACTIONS] [REQUIRED]
Base: if yes at Q_ParentGuardianStatus

Please indicate how your interactions with your child(ren) have been impacted by the COVID-19 pandemic.
(Please select one option on each row)

Commented [HZ1]:

[COLUMNS] [ANCHOR]
More
Less
No change
Not applicable
Prefer not to say

[ROWS] [RANDOMIZE]
Having quality time with my child(ren)
Feeling closeness with my child(ren)
Showing love or affection to my child(ren)
Observing resilience (strength and perseverance) in my child(ren)
Disciplining my child(ren)
Conflicts with my child(ren)
Using harsh words with my child(ren)
Yelling/shouting at my child(ren)

TAB NOTE:
Detail Table Requirements

[QUESTION TYPE: single choice **LAYOUT:** default (kato)**]**
[Q_CHILDVACCINE_OVER12] [REQUIRED]
Base: If 'Yes' at Q_ParentGuardianStatus

If you have a child(ren) age 12 to 18, have they been vaccinated?

[ANSWERS] [ANCHOR]
Yes
No
Prefer not to answer
Does not apply

TAB NOTE:
Detail Table Requirements

maru/matchbox

[QUESTION TYPE: single choice LAYOUT: default (kato)]
[Q_CHILDVACCINE_UNDER12] [REQUIRED]
Base: if yes at Q_ParentGuardianStatus

If you have a child(ren) under the age of 12, will you get them vaccinated?

[ANSWERS] [ANCHOR]

Yes
No
Have not yet decided
Prefer not to answer
Does not apply

TAB NOTE:
Detail Table Requirements

[QUESTION TYPE: multi choice LAYOUT: default (kato)]
[Q_CHILDVACCINEBARRIERS] [REQUIRED]
Base: if yes at Q_ParentGuardianStatus

Are any of the following barriers to your child(ren) being vaccinated?

[ANSWERS] [ANCHOR]
Not confident in the safety of a vaccine for my child(ren)
Concern about risks and side effects for my child(ren)
Will wait until it feels safe to get a vaccine for my child(ren)
Do not consider it necessary for my child(ren) to get a vaccination
Do not believe in vaccination for my child(ren)
Have not yet decided
Other reason
My child(ren) have a pre-existing medical condition
My child(ren) have already had or I think they have had COVID-19
My child is hesitant about getting the vaccine

TAB NOTE:
Detail Table Requirements

[QUESTION TYPE: open end LAYOUT: text field (single line) narrowtextbox - numeric (kato)]
[QHOUSEHOLD] [REQUIRED]
Base: Total

How many people reside in your household?

[RANGE: 1 TO 99]

maru/matchbox

TAB NOTE:
Detail Table Requirements

[QUESTION TYPE: multi choice **LAYOUT:** default (kato)**]**
[Q_HOUSEHOLDLIVING] [REQUIRED]
Base: Total

Which of the following best describes your living arrangements? (Please select all that apply)

[ANSWERS] [ANCHOR]
I live alone
Living with a spouse or partner
Living with friend(s) or housemate(s)
Living with siblings
Living with my child(ren) who are over 18
Living with my child(ren) who are under 18
Living with other adult family members (e.g., parents, grandparents)
Living with grandchildren
Other
Prefer not to answer **[EXCLUSIVE]**
None of the above **[EXCLUSIVE]**

TAB NOTE:
Detail Table Requirements

[QUESTION TYPE: text instruction**]**
[Q_FINAL] [REQUIRED]
Base: Total

Thank you for taking part in this survey. If you've been affected by this topic and would like any more information, need advice, or support, you can go to the following place for help:
Canadian Mental Health Association

Please click forward to complete the survey...

[PLEASE ADD THE FOLLOWING RE-DIRECT LINK AT THE END OF SURVEY TO "https://cmha.ca/"]

MVC Sample Demos to Append:

- Gender
- Age
- Province
- Urban/Rural
- HHI
- Education
- Household Size (HH Size)
- Community Size (PPO_CNTR_POP_SIZE_GROUP)
- QWave Variable (Wave 1,Wave 2, Wave 3 and Wave 4)
